# Quantification of the ischemic burden on cardiac magnetic resonance perfusion maps by perfusion threshold analysis

**DOI:** 10.1186/1532-429X-15-S1-P202

**Published:** 2013-01-30

**Authors:** Amedeo Chiribiri, Niloufar Zarinabad, Gilion Hautvast, Shazia T Hussain, Boris Bigalke, Andreas Schuster, Marcel Breeuwer, Eike Nagel

**Affiliations:** 1 King's College, London, UK; 2 Philips Healthcare, Best, Netherlands; 3 Eindhoven University of Technology, Eindhoven, Netherlands; 4 Philips Innovation Group - Healthcare Incubators, Eindhoven, Netherlands

## Background

High-resolution quantitative analysis of myocardial blood flow (MBF) by cardiac magnetic resonance (CMR) perfusion has been recently proposed and validated in preclincal studies. It aims to combine the advantages of both visual assessment and quantitative analysis in a single test. However, incorrect registration between stress and rest images can hamper myocardial perfusion reserve (MPR) calculation from high-resolution perfusion maps. The aim of this study was to test the feasibility of MPR analysis from high-resolution perfusion maps as well as to test MBF threshold analysis, a novel analysis approach capable to measure the extent of ischemic myocardium with high spatial accuracy.

## Methods

Ten patients with single-vessel coronary artery disease (CAD) and ten control subjects were analyzed at 3 Tesla. Perfusion data were analyzed on high-resolution maps and at segmental level to simulate MPR for variable degrees of image misregistration. MBF threshold analysis was then applied to high-resolution perfusion maps. In analogy to one of the methods currently in use for the quantification of late gadolinium enhancement, pixels with abnormally reduced coronary flow reserve were defined on the basis of the average MBF in areas with visually normal perfusion. In this exploratory analysis, the ischemic myocardium was defined as pixels with MBF reduced below the 90th, 95th and 99th percentile of the average epicardial MBF of the reference segment. MBF threshold analysis defines the amount of ischemic myocardium as a percentage of the LV (global ischemic burden) or of a perfusion territory (territorial ischemic burden).

## Results

MPR analysis was hampered on high-resolution perfusion maps by high levels of error due to image misregistration (ranging from 25% to 41%), compared with significantly lower error on segmental analysis (0.1 to 7.7% for corresponding degrees of image misregistration; p<0.0001).

Conversely, MBF threshold analysis was able to identify the presence and the location of CAD in global and segmental LV analysis (p<0.001).

The normal distribution of the MBF results on high-resolution maps was confirmed by the P-P plot and the results of the skewness test, which was on average 1.1±0.2.

Different thresholds of MBF identified areas of abnormally reduced MBF on the high-resolution perfusion maps, with an extension proportional to the absolute value of each threshold.

## Conclusions

Without a excellent consistency of image registration MPR analysis is not feasible on high-resolution perfusion maps due to the considerable error caused even by minimal degrees of image misregistration. On the contrary, MBF threshold analysis is capable to identify the presence of CAD and to identify the diseased coronary artery segment.

## Funding

The authors acknowledge financial support from the Department of Health via the National Institute for Health Research (NIHR) comprehensive Biomedical Research Centre award to Guy's & St Thomas' NHS Foundation Trust in partnership with King's College London and King's College Hospital NHS Foundation Trust. The Centre of Excellence in Medical Engineering funded by the Wellcome Trust and EPSRC under grant number WT 088641/Z/09/Z. Funded by the British Heart Foundation award RE/08/003.

